# Incidental Discovery of Klippel-Feil Syndrome: Beyond the Expected Clinical Triad

**DOI:** 10.7759/cureus.58538

**Published:** 2024-04-18

**Authors:** Ana L Melero-Pardo, Tatiana C Pimentel-Soler, Dalvert H Polanco, Janelly Simons-Obregon, Joelia Blanco-Colón, Carlos R Benitez-Colón

**Affiliations:** 1 Medicine, Universidad Central del Caribe School of Medicine, Bayamon, PRI; 2 Internal Medicine, Universidad Central del Caribe School of Medicine, Bayamon, PRI

**Keywords:** sprengel deformity, congenital malformations, scoliosis, renal agenesis, klippel-feil syndrome

## Abstract

Klippel-Feil syndrome (KFS) is a rare congenital disorder characterized by the fusion of cervical vertebrae, limiting neck mobility, and often presenting with clinical manifestations such as neck pain, stiffness, and neurological deficits. While the classical presentation of KFS includes a "clinical triad" comprising a shortened neck, a low posterior hairline, and limited cervical motion, not all patients exhibit all three features. This case report presents an 81-year-old male with the complete KFS triad and underscores the diagnostic challenges and management strategies associated with this condition. Despite the rarity of KFS, understanding it is crucial for clinicians due to its profound implications on patient management and quality of life. This case emphasizes the importance of clinical suspicion in Internal Medicine, showcasing how an isolated presentation may often be a manifestation of an underlying congenital condition.

## Introduction

Klippel-Feil syndrome (KFS) is a rare congenital disorder characterized by the fusion of two or more cervical vertebrae. This fusion results in a limited range of motion in the neck, leading to various clinical manifestations such as neck pain, stiffness, and neurological deficits [1]. The syndrome was first described by Maurice Klippel and André Feil in 1912 and is estimated to occur in approximately 1 in 40,000 live births [2]. It predominantly affects females, with a prevalence of approximately 60% [3]. KFS is believed to result from genetic mutations in specific genes: growth differentiation factor 6 or 3 (GDF6 or GDF3), which are part of the transforming growth factor beta (TGF-β) protein family, and mesenchyme homeobox 1 (MEOX1) [4,5]. While GDF6 is linked to bone and cartilage growth, GDF3 influences ocular and skeletal development. MEOX1 is crucial for somitogenesis, and mutations in this gene have been found to produce Klippel-Feil-like characteristics in mice [4,5].

The clinical presentation of KFS can vary widely, ranging from asymptomatic cases discovered incidentally on radiographic imaging to severe cases with significant neurological complications [6-9]. Clinically, KFS is often linked with a "clinical triad" which includes a shortened neck, a low hairline at the back, and limited cervical motion [1]. Nevertheless, research indicates that fewer than half of KFS patients display all three characteristics [10]. Common associated anomalies include scoliosis, renal abnormalities, and cardiac defects [11,12]. Diagnosis is typically made based on clinical evaluation, radiographic findings, and sometimes confirmed with genetic testing.

Despite its rarity, understanding KFS is crucial for clinicians as it can have profound implications on patient management and quality of life. This report details the case of an 81-year-old male exhibiting the complete triad of Klippel-Feil syndrome and explores our diagnostic approach and associated anomalies.

## Case presentation

**Figure 1:**
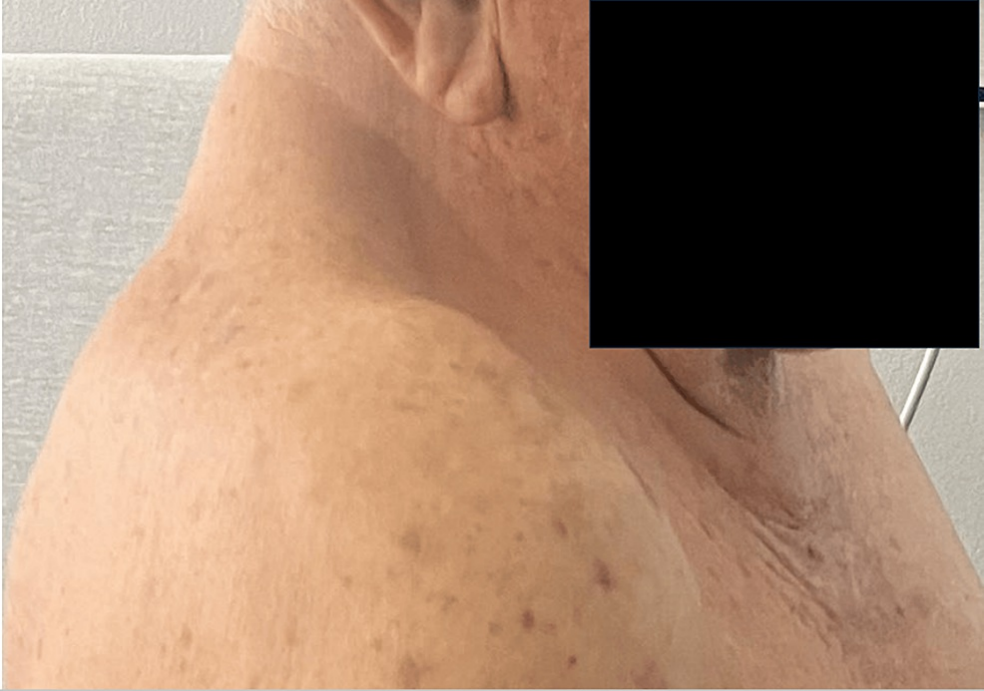
Photograph illustrating a lateral view of the patient's shortened neck characteristic of Klippel-Feil syndrome. Lateral photograph highlighting the characteristic shortened neck observed in a patient with Klippel-Feil syndrome. Identity obscured for privacy with a black box.

**Figure 2:**
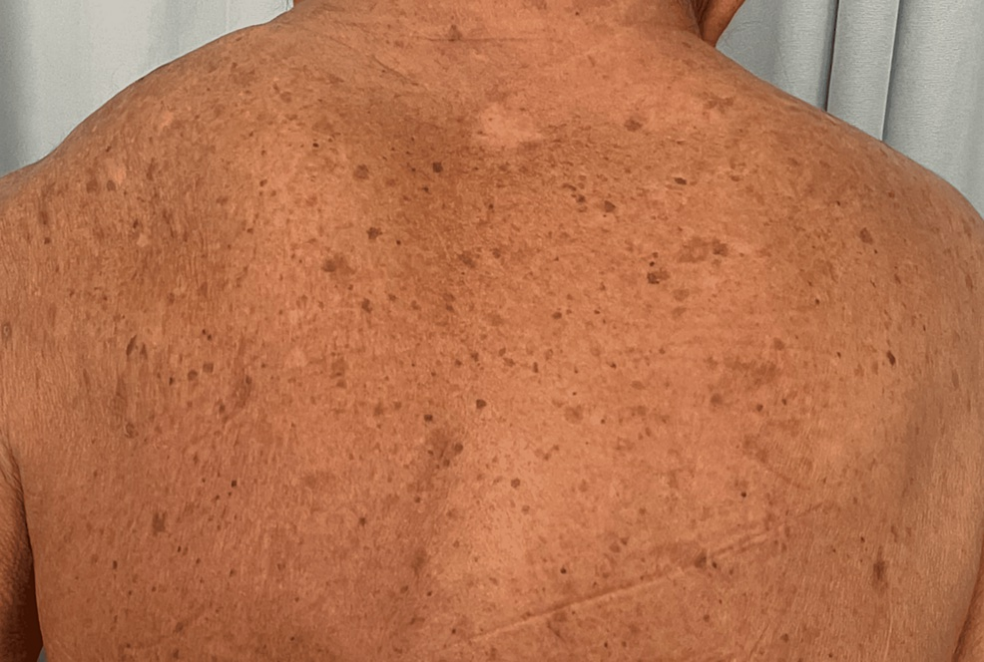
Photograph of the patient’s back demonstrates an elevated left scapula known as Sprengel deformity.

**Figure 3:**
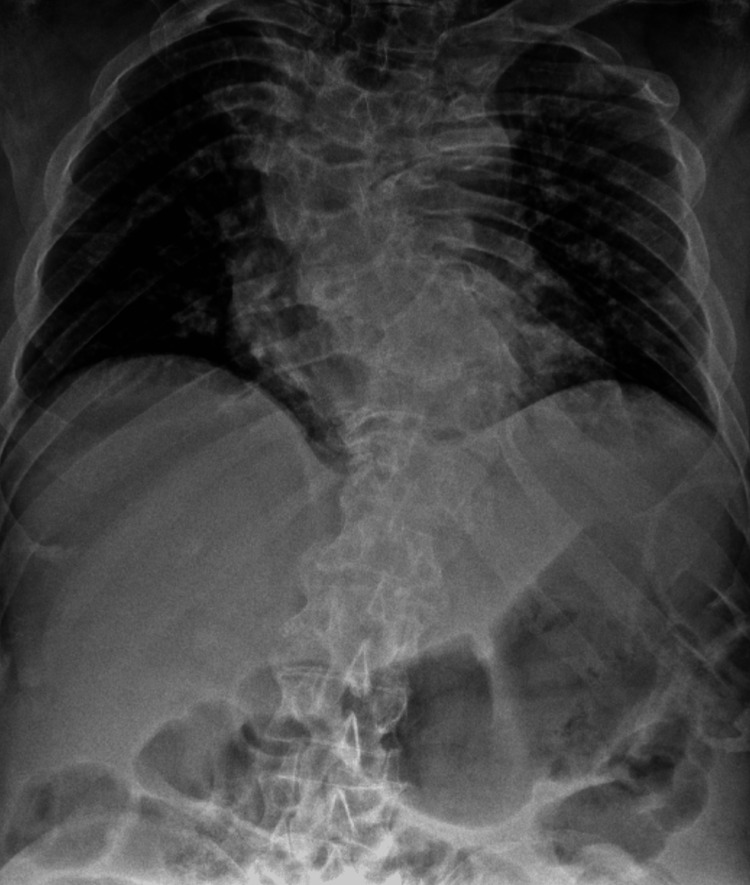
Chest X-ray demonstrating thoracolumbar scoliosis.

An 81-year-old male with a medical history significant for arterial hypertension, scoliosis, and left renal agenesis presented to the emergency department after experiencing a fall from standing height. The patient reported that he had tripped over his own feet, resulting in his back hitting the ground. Following the fall, he developed bilateral leg weakness, rendering him unable to walk. He also reported profuse sweating, fatigue, incoherent speech, shortness of breath, and chills. The patient mentioned a history of chronic imbalance but denied experiencing nausea, vomiting, fever, palpitations, loss of consciousness, loss of sphincter control, or recent exposure to sick contacts. His past surgical history included inguinal herniorrhaphy and bilateral cataract surgery. He is a father to three offspring, comprising two males and one female. Notably, both male offspring have intellectual disabilities. Upon further review of the systems, the patient reported symptoms of lower extremity edema, back pain, and weakness in the lower extremities. Physical examination revealed a patient with a short stature, short neck, limited range of motion in the neck, and evident high left scapula, known as Sprengel deformity (Figures 1, 2). Chest X-ray demonstrated thoracolumbar scoliosis (Figure 3).

**Figure 4:**
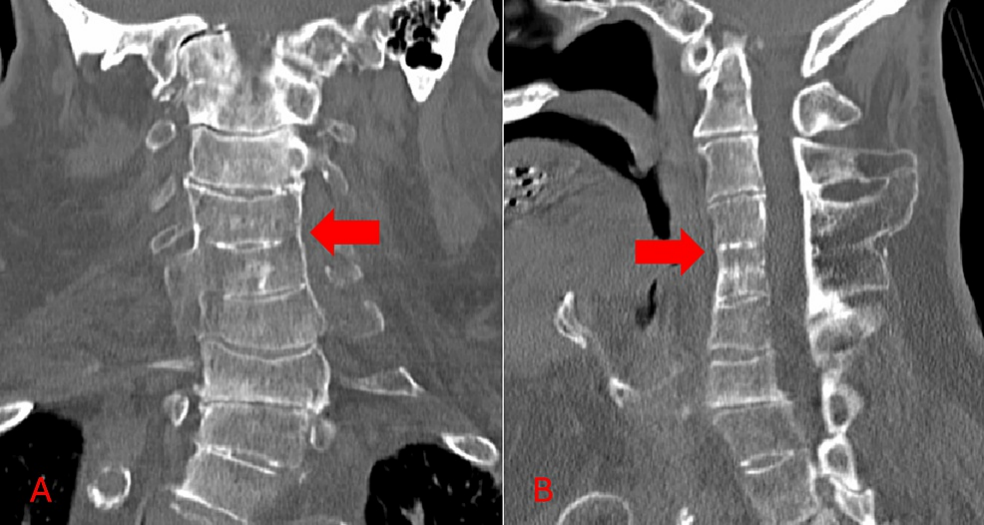
Coronal (A) and sagittal (B) CT scans of the cervical spine in Klippel-Feil syndrome. CT scans were conducted without contrast. Fusion between C3-C4 and deformities affecting several cervical vertebrae are evident. Red arrows in both panels highlight the fusion.

**Figure 5:**
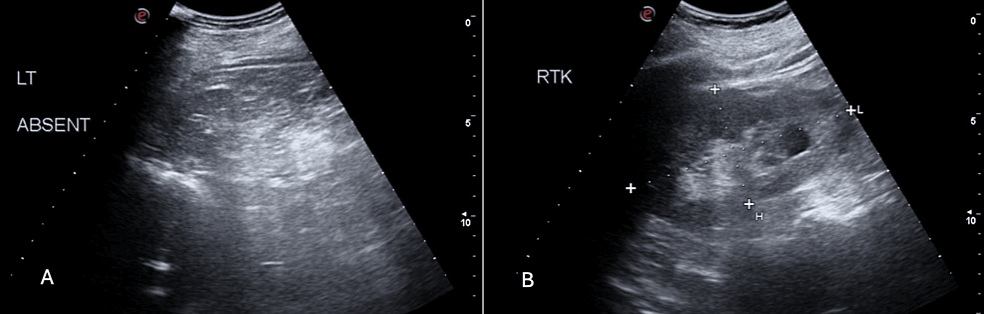
Renal ultrasound demonstrating unilateral renal agenesis. Panel A demonstrates the absence of the left kidney, indicative of unilateral renal agenesis. Panel B displays the normal appearance of the right kidney.

A computed tomography (CT) scan without contrast of the cervical spine revealed fusion involving cervical vertebrae C3-C4 (Figures 4A, 4B). A thoracic spine CT without contrast showed incomplete fusion of the spinous process at C7 level. Renal ultrasound demonstrated unilateral renal agenesis (Figures 5A, 5B).

## Discussion

This case highlights the importance of maintaining a high level of clinical suspicion in Internal Medicine, especially when facing patients whose symptoms may appear unrelated. While the patient initially presented to the emergency department following a fall, a comprehensive evaluation revealed underlying KFS, a rare congenital disorder characterized by cervical vertebral fusion. This case demonstrates that an isolated clinical presentation may often be a manifestation of an underlying systemic or congenital condition.

KFS is known to manifest with a wide spectrum of clinical features, including but not confined to, limited neck mobility, scoliosis, and various associated anomalies such as renal agenesis, Sprengel deformity, and intellectual disabilities. In the literature, the incidence of complications related to congenital urogenital anomalies in Klippel-Feil syndrome ranges from 25% to 35%, with unilateral renal agenesis being the most common [13,14]. In our patient, a history of unilateral renal agenesis was noted as an incidental finding from the sonographic evaluation. Furthermore, our patient exhibited a noticeable elevation of the left scapula during the physical examination, consistent with Sprengel deformity, appearing in 7-24% of individuals with KFS. This deformity leads to visible changes in the upper limb's appearance and potential muscle function issues [15]. 

In this case, the patient's history of scoliosis, short stature, short neck, and limited neck range of motion were indicative of possible musculoskeletal abnormalities. The diagnosis of KFS in this patient was confirmed through imaging studies, specifically a CT scan of the cervical spine, which demonstrated characteristic vertebral fusion in C3-C4 and thoracic spine CT incomplete fusion of the spinous process at C7. This diagnosis was particularly important as it not only explained the patient's chronic musculoskeletal symptoms but also provided valuable insights into the etiology of his current presentation following the fall. The finding of bilateral leg weakness and the patient's reported chronic imbalance could be attributed to neurological complications associated with KFS. Patients with KFS are prone to cervical cord injury after a minor fall or a traumatic episode [16,17].

The importance of clinical suspicion in Internal Medicine cannot be overstated, as it guides clinicians to consider a broad differential diagnosis and order appropriate diagnostic tests to confirm or rule out potential underlying conditions. In this case, a thorough evaluation beyond the immediate presenting complaint of a fall led to the discovery of an underlying congenital condition that had likely contributed to the patient's overall health and well-being.

## Conclusions

This case underscores the imperative role of clinical expertise and a comprehensive assessment in Internal Medicine. Given the specific challenges posed by Klippel-Feil syndrome (KFS), including its wide-ranging clinical manifestations and potential neurological implications, there is a heightened importance in this population. The presentation of an 81-year-old male with the complete triad of KFS, following a seemingly unrelated fall, emphasizes that an isolated clinical event can often be a clue to an underlying congenital or systemic disorder. Considering the falling prevalence in similar demographics, early recognition and diagnosis of KFS are even more pivotal. Not only do they explain chronic musculoskeletal symptoms, but they also inform management strategies and help understand potential complications. As clinicians, maintaining a high index of suspicion and pursuing thorough evaluations beyond immediate complaints are essential to ensure optimal patient care and outcomes. This case serves as a reminder for clinicians to delve deeper into the broader differential diagnosis, thereby facilitating the discovery and management of underlying conditions that may significantly impact a patient's health and quality of life.
